# High-resolution prediction models for *Rhipicephalus microplus* and *Amblyomma cajennense* s.l. ticks affecting cattle and their spatial distribution in continental Ecuador using bioclimatic factors

**DOI:** 10.1007/s10493-023-00883-3

**Published:** 2024-02-23

**Authors:** Ximena Pérez-Otáñez, Richar Rodríguez-Hidalgo, Sandra Enríquez, Maritza Celi-Erazo, Washington Benítez, Claude Saegerman, Franklin Vaca-Moyano, Lenin Ron-Garrido, Sophie O. Vanwambeke

**Affiliations:** 1 Instituto de Investigación en Zoonosis-CIZ, Universidad Central del Ecuador, Quito, Ecuador; 2 Facultad de Medicina Veterinaria y Zootecnia, Universidad Central del Ecuador, Quito, Ecuador; 3 Facultad de Ciencias Agrícolas, Universidad Central del Ecuador, Quito, Ecuador; 4 Center for Earth and Climate Research, Earth & Life Institute, Université Catholique de Louvain-UCLouvain, Louvain-La-Neuve, Belgium; 5 Research Unit of Epidemiology and Risk Analysis Applied to Veterinary Science (UREAR-ULiège), Fundamental and Applied Research for Animals & Health (FARAH) Center, Faculty of Veterinary Medicine, University of Liege, Liège, Belgium

**Keywords:** Distribution model, Suitability, Bioclimatic, Cattle ticks, Ecuador, *Rhipicephalus microplus*, *Amblyomma cajennense* s.l.

## Abstract

In Ecuador, the main tick species affecting cattle are *Rhipicephalus microplus* and *Amblyomma cajennense* sensu lato. Understanding their spatial distribution is crucial. To assess their distribution, data from 2895 farms visited between 2012 and 2017 were utilized. Ticks were collected during animal inspections, with each farm’s location georeferenced. Bioclimatic variables and vapor pressure deficit data were obtained from Climatologies at High resolution for the Earth´s Land Surface Areas (CHELSA) dataset. They were overlaid to develop predictive maps for each species using Random Forest (RF) models. The cross-validation results for RF prediction models showed high accuracy for both *R. microplus* and *A. cajennense* s.l. presence with values of accuracy = 0.97 and 0.98, sensitivity = 0.96 and 0.99, and specificity = 0.96 and 0.93, respectively. A carefully selected subset of bioclimatic variables was used to describe the presence of each tick species. Higher levels of precipitation had positive effect on the presence of *R. microplus* but a negative effect on *A. cajennense* s.l. In contrast, isothermality (BIO3) was more important for the presence of *A. cajennense* s.l. compared to *R. microplus.* As a result, *R. microplus* had a broader distribution across the country, while *A. cajennense* s.l. was mainly found in coastal areas with evident seasonality. The coexistence of both species in some regions could be attributed to transitional zones, whereas high altitudes limited tick presence. This information can aid in developing appropriate tick management plans, particularly considering *A. cajennense* s.l.’s broad host range species and *R. microplus*’s specificity for cattle. Moreover, the predictive models can identify areas at risk of associated challenging hemoparasite, requiring special attention and mitigation measures.

**Graphic abstract:**

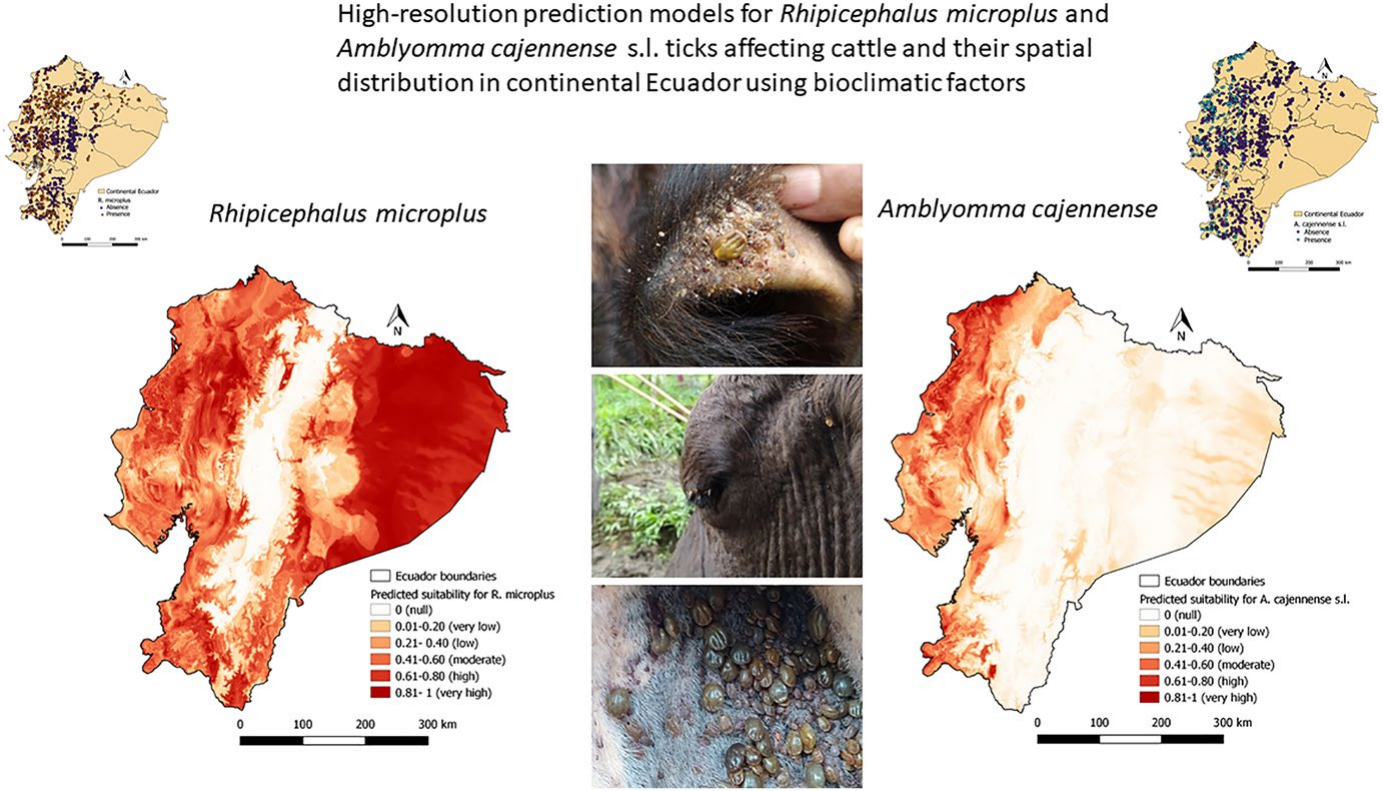

## Introduction

Ecuador is a megadiverse country with particular climatic characteristics due to the Andes mountain range. The agricultural sector is important for the economy, accounting for 7.7% of the gross domestic product (Primicias 2022). Around 70% of livestock activities take place in tropical and subtropical zones (Guillén and Muñoz 2013), but has been affected by the presence of ticks. Hard ticks of the Ixodidae family affect mammals. Cattle in Ecuador are frequently affected by ticks, and humans occasionally. These ectoparasites cause substantial economic losses in the livestock sector (Paucar-Quishpe et al. 2023). Widespread acaricide resistance has been identified, causing additional control challenge and loss (Pérez-Otáñez et al. 2023).

The main species affecting cattle in Ecuador are *Rhipicephalus microplus* and *Amblyomma cajennense* s.l. (Bustillos and Rodríguez 2016; Maya-Delgado et al. 2020; Paucar et al. 2022). *R. microplus* is widely distributed in tropical and subtropical areas of Australia, Africa, and Latin America (doubtfully established in Chile) (González-Acuña and Guglielmone 2005). It arrived around five centuries ago with the Spanish colonizers (Estrada-Peña 1999) and adapted well to Ecuador. *A. cajennense* s.l. is distributed from the southern USA to northern Argentina, and it is a multi-host ectoparasite in mammals including humans incidentally. These species are not controlled satisfactorily in many countries (Lima et al. 2000; Forero-Becerra et al. 2022) and cause direct and indirect losses (Alcala-Canto et al. 2018). In cattle, parasitism results in weight loss, reduction in milk production (Estrada-Peña et al. 2006b) estimated at around 90.2 L per cow per year (Marques et al. 2020), and weakness due to blood loss. Ticks also vector pathogens that affect cattle. *R. microplus* is an efficient vector for *Babesia bovis* and *Babesia bigemina*, and a suspected vector for *Anaplasma* spp. in Ecuador (Escobar et al. 2015; Insuaste Taipe 2021), *A. cajennense* s.l. is also known to transmits the bacterium *Ehrlichia ruminantium*, which has not yet been reported in Ecuador. For the farmers, costs are associated to control and morbidity as well as animal mortality (Alonso-Díaz et al. 2013; Pothmann et al. 2016; Kasaija et al. 2021). In addition, Paucar-Quishpe et al. (2023) associated cattle financial losses with tick acaricide-resistances in Ecuador.

The survival and development of cattle ticks depend on climatic and management conditions. While hosts are necessary for tick presence, we expect cattle tick survival and development to be affected by bioclimatic variables such as rainfall and temperature, that affect the number of generations per year, and thus, abundance. The most limiting factors for their presence, as well as the tick ecotype, can vary between regions (Lima et al. 2000; Estrada-Peña 2023) (Lima et al. 2000; Estrada-Peña et al. 2006b). At broad scales, climate factors have been found useful to delimit tick distribution (Estrada-Peña et al. 2006a, b).

To develop adequate control plans, it is necessary to understand the geographical distribution, and environmental suitability for ticks (Estrada-Peña 1999). Although both *R. microplus* and *A. cajennense* s.l. affect cattle, their biology differs. *R. microplus* fulfills its biological cycle on one host, and has high host specificity for cattle (Nava et al. 2013). *A. cajennense* s.l. fulfills its cycle on three hosts, infesting mainly equines (Alonso-Díaz et al. 2013).Current on-farm tick control often neglects essential aspects of tick biology and species-specific traits, for example both species, *Amblyomma cajennense* s.l. and *Rhipicephalus microplus*, exhibit unique patterns of acaricide resistance, requiring separate resistance analyses and rotation schedules. *A. cajennense* s.l. may complete early life stages in the wild, emphasizing the need to clear vegetation around pastures. For *R. microplus*, pasture height and type management is essential due to its life cycle. Manually removing ticks daily could be effective for *R. microplus* population control, but *A. cajennense* s.l.’s longer hypostome could damage skin cattle. Attention to these distinct characteristics is vital for effective tick control strategies. Application of control measures without consideration for tick biology can lead to poor acaricide efficacy and induce acaricide resistance which has been widely reported for *R. microplus* in Ecuador (Rodríguez-Hidalgo et al. 2017; Maya-Delgado et al. 2020; Dzemo et al. 2022; Pérez-Otáñez et al. 2023; Paucar-Quishpe et al. 2023). Only a few studies have addressed acaricide resistance in *A. cajennense* s.l., but resistance is probable as well (Alonso-Díaz et al. 2013).

Various distribution models have been published in the past for *R. microplus* in South America and West Africa (Estrada-Peña 1999; Estrada-Peña et al. 2006c; De Clercq et al. 2015; Zannou et al. 2022). They mostly use data drawn from bibliographic sources that often do not represent well the Andes and the particular climatological conditions present in Ecuador. Estrada-Peña (1999) found no suitability for *R. microplus* in Ecuador. Estrada-Peña et al. (2005), using updated data and a different methodology, found suitability in few parts of the Coastal zone. Marques et al. (2020) in their distribution model of *R. microplus* found a high suitability in the Andean zone of Ecuador. As for *A. cajennense* s.l. there are few distribution prediction studies in South América including Ecuador. Aguilar-Domínguez et al. (2021) showed suitability in the occidental part of the Coastal zone of Ecuador for *Amblyomma mixtum*, a member of the *A. cajennense* complex, similar than Estrada-Peña et al. (2014).

This study utilized data from “Climatologies at High resolution for the Earth’s Land Surface Areas” (CHELSA). CHELSA provides high-resolution bioclimatic variables over the Earth’s land surfaces at a 1 km-resolution suitable for broad scale modelling of tick climatic suitability. The precision provided by CHELSA increases the accuracy of predictive models (Karger et al. 2017). Greater model accuracy could support targeted tick management strategies and contributes to the containment of tick-borne diseases.

Thus, the present study aims to update the distribution cattle ticks (*R. microplus* and *A. cajennense* s.l.) in Ecuador, considering that their presence in highlands remain rare (Chávez-Larrea et al. 2021), and international databases may not adequately capture the specificities of mountain conditions in the Andes. Therefore, the main objective of this study is to use an extensive national dataset of presence and absence of ticks on cattle (*R. microplus* and *A. cajennense* s.l.) to model associations with bioclimatic variables. Furthermore, we predict suitable areas where these ticks can affect cattle in continental Ecuador. The findings of this study will provide valuable information for future prevention and control plans.

## Methods

### Study area

**Fig. 1:**
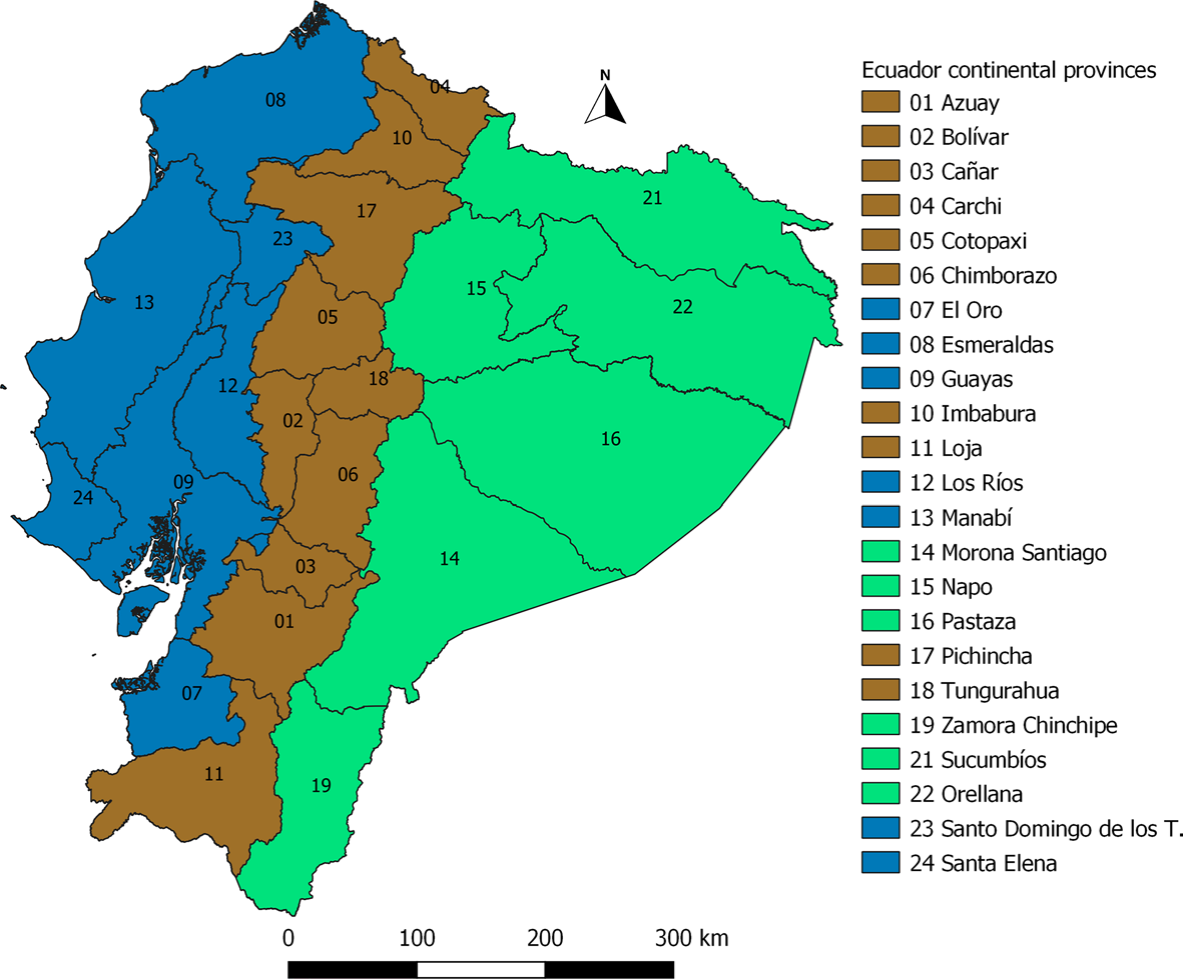
Continental Ecuadorian provinces

We assembled several cross-sectional studies carried out between 2012 and 2017 by the Instituto de Investigación en Zoonosis (CIZ) in Universidad Central del Ecuador (UCE). Specifically, the following studies were used: the “National survey about bovine Brucellosis, tuberculosis, and cattle ticks” (Maya-Delgado et al. 2020; Paucar et al. 2021), the “Spatial analysis and ecological-epidemiological aspects of *Rhipicephalus microplus* infestation and its resistance to acaricides”, the “Wild arthropod vectors and domestic reservoirs as indicators of vulnerability to re-emerging zoonotic diseases in the Ecuadorian Amazon", and the "Molecular epidemiology of parasites and microorganisms of zoonotic interest: cattle screwworm and ticks". Altogether, ticks were sampled from 22 of the 23 Ecuador continental provinces. In each farm three bovines selected randomly were sampled for ticks. 2895 cattle farms were visited in the three continental regions of the country. One Andean province (Cañar) was not included in any sample, as it has relatively fewer cattle farms. Table 1 and Fig. 1 describe the sampling in the provinces. Geographical coordinates were recorded in each farm with a Garmin GPS (WGS 84).

**Table 1:** Number of farms sampled in each province

Region	Provinces	Provinces number	Farms
Andean	Azuay	01	142
Andean	Bolivar	02	241
Andean	Carchi	04	81
Andean	Chimborazo	06	276
Andean	Cotopaxi	05	220
Andean	Imbabura	10	144
Andean	Loja	11	332
Andean	Pichincha	17	127
Andean	Tungurahua	18	199
Coastal	El Oro	07	86
Coastal	Esmeraldas	08	98
Coastal	Guayas	09	137
Coastal	Los Rios	12	110
Coastal	Manabí	13	312
Coastal	Santa Elena	24	17
Coastal	Sto. Domingo De Los Tsáchilas	23	111
Amazon	Morona Santiago	14	7
Amazon	Napo	15	79
Amazon	Orellana	22	9
Amazon	Pastaza	16	52
Amazon	Sucumbíos	21	30
Amazon	Zamora Chinchipe	19	85
Total		2895

Tick collection and morphological identification.

The ticks collected were stored in tubes with absolute ethanol (100%) and taken to the Unidad de Entomología Aplicada of Instituto de Investigación en Zoonosis (CIZ). Ticks were morphologically identified using an Olympus SZ51 stereomicroscope with magnifications × 0.8–4.5 and taxonomic keys (Guerrero 1996; Voltzit 2007; Nava et al. 2014).Ticks were identified to genus and species level. We focused on *R. microplus* and *A. cajennense* s. l., as they were the most abundant (Rodríguez-Hidalgo et al. 2017; Maya-Delgado et al. 2020; Paucar et al. 2022).

***Ethical considerations.***

The attainment of the study’s objectives did not necessitate approval from research ethics committees, as the experimental procedures involved an unregulated invertebrate species.

### Bioclimatic data

We used the 19 bioclimatic layers from CHELSA Bioclim, derived from the monthly mean, max, mean temperature, and mean precipitation values and the vapor pressure deficit over 1981–2010 (Table 2). This information is available at a horizontal resolution of 30 arc sec. Although relative humidity has been commonly used, vapor pressure deficit (VPD) is a more useful parameter for evaluating environmental conditions in relation to tick development, as it accounts for the air’s drying power (Wollaeger and Runkle 2015; Pascoe et al. 2019; Estrada-Peña 2023). The VPD data was calculated from hurs, considered a unitless fraction, and tas in ◦C as with this formula: VPD = *e*_sat_(tas) × (1 − hurs)

$$\text{VPD }= {e}_{{\text{sat}}}\left({\text{tas}}\right)\times \left(1-{\text{hurs}}\right),$$

where hurs is the relative humidity, *e*_sat_(tas) is the saturation vapor pressure. To approximate *e*_sat_(tas), the Magnus equation was used with the coefficients of Sonntag (1990):

$${e}_{{\text{sat}}}\left({\text{tas}}\right)=0.6112\times {\text{e}}\frac{17.62-tas}{\left(243.12+tas\right)}.$$

**Table 2:** Bioclimatic variables obtained from CHELSA climatologies at high resolution for the Earth’s land surface areas

Short name	Long name	Unit	Explanation
Bio1	Mean annual air temperature	°C	Mean annual daily mean air temperatures averaged over 1 year
Bio2	Mean diurnal air temperature range	°C	Mean diurnal range of temperatures averaged over 1 year
Bio3	Isothermality	°C	Ratio of diurnal variation to annual variation in temperatures
Bio4	Temperature seasonality	°C/100	Standard deviation of the monthly mean temperatures
Bio5	Mean daily maximum air temperature of the warmest month	°C	Highest temperature of any monthly daily mean maximum temperature
Bio6	Mean daily minimum air temperature of the coldest month	°C	Lowest temperature of any monthly daily mean maximum temperature
Bio7	Annual range of air temperature	°C	Difference between the Maximum Temperature of warmest month and the minimum temperature of coldest month
Bio8	Mean daily mean air temperatures of the wettest quarter	°C	Wettest quarter of the year is determined (to the nearest month)
Bio9	Mean daily mean air temperatures of the driest quarter	°C	Driest quarter of the year is determined (to the nearest month)
Bio10	Mean daily mean air temperatures of the warmest quarter	°C	Warmest quarter of the year is determined (to the nearest month)
Bio11	Mean daily mean air temperatures of the coldest quarter	°C	Coldest quarter of the year is determined (to the nearest month)
Bio12	Annual precipitation amount	kg m^−2^ year^−1^	Accumulated precipitation amount over 1 year
Bio13	Precipitation amount of the wettest month	kg m^−2^ month^−1^	Precipitation of the wettest month
Bio14	Precipitation amount of the driest month	kg m^−2^ month^−1^	Precipitation of the driest month
Bio15	Precipitation seasonality	kg m^−2^	Coefficient of Variation is the standard deviation of the monthly precipitation estimates expressed as a percentage of the mean of those estimates (i.e. the annual mean)
Bio16	Mean monthly precipitation amount of the wettest quarter	kg m^−2^ month^−1^	Wettest quarter of the year is determined (to the nearest month)
Bio17	Mean monthly precipitation amount of the driest quarter	kg m^−2^ month^−1^	Driest quarter of the year is determined (to the nearest month)
Bio18	Mean monthly precipitation amount of the warmest quarter	kg m^−2^ month^−1^	Warmest quarter of the year is determined (to the nearest month)
Bio19	Mean monthly precipitation amount of the coldest quarter	kg m^−2^ month^−1^	Coldest quarter of the year is determined (to the nearest month)
VPD_max	Maximum monthly vapor pressure deficit	Pa	Highest monthly vapor pressure deficit
VPD_mean	Mean monthly vapor pressure deficit	Pa	Average monthly vapor pressure deficit over 1 year
VPD_min	Minimum monthly vapor pressure deficit	Pa	Lowest monthly vapor pressure deficit

VPD was calculated in R, using the package bigleaf (Brun et al. 2022). The area of continental Ecuador was extracted using the “mask” function from “raster” package under R environment. Each bioclimatic raster variable, as CHELSA recommends, was multiplied for the scale value and then added to the offset value (Karger et al. 2017).

### Correlative analysis

In order to identify the strongest predictors of tick presence, generalized linear models (GLM) analyses were conducted using presence and absence data on *R. microplus*, and *A. cajennense* s.l., and as explanatory variables, the climatology data Bio1 to Bio19 and the vapor pressure deficit mean, minimum, and maximum. The "logit" function, part of the logistic model, was chosen as the link function for regression due to its preference for natural interpretations of coefficients in terms of odds ratios. The logit model is favored over the probit model because the interpretation of betas in probit regression is less intuitive. Although a comparison of both models based on their likelihood values could have been conducted, our preference was for the logit model to facilitate the interpretation of parameters and measurement of their effects. For the multivariable analyses, we included the explanatory variables with a P-value < 0.2 in the univariate analyses. Effect of collinearity in the model was reduced by removing variables presenting a variance inflation factor (VIF) higher than 8, with the exception of the variables known from literature to be important in tick biology. BIO1, the mean annual temperature, is associated to more favorable conditions for tick development Higher mean annual temperature shortens life cycle, lengthen seasonal activity (Alcala-Canto et al. 2018; Pascoe et al. 2019). Since all variables are derived from temperature and precipitation, they are expected to be collinear. Forward stepwise selection was used to build the final multiple GLM model, with the stepAIC function into the “MASS” package. We used the subset of variables producing the lowest Akaike information criteria (AIC). Finally, we computed the sensitivity, specificity, and area under the receiver operating characteristic (ROC) curve (AUC-ROC), using the “CARET” and “Proc” R packages. The bioclimatic variables were standardized using “scale” function in R.

### Predictive models for cattle tick’s habitat suitability

Random Forest (RF), a supervised model, was used to model habitat suitability of cattle ticks (Kopsco et al. 2022; Zannou et al. 2022). Using the presence and absence data and the results of the multiple GLM, the habitat suitability prediction model in continental Ecuador under bioclimatic conditions was modelled using the “Random Forest” package in R.

RF yields a value from 0 (completely unsuitable) to 1 (fully suitable). Using the "importance" function in the “Random Forest” package, we calculated the Mean Square Error (MSE), revealing elevated percentages for the most significant variables within the resulting RF model. The models were evaluated using sensitivity and specificity and area under the ROC curve (AUC-ROC). The models were trained with 80% of the data (randomly selected), and validated with the other 20% for 10 times, to obtain the mean sensitivity, specificity, and accuracy. Models were adjusted for: *R. microplus*, and *A. cajennense* s.l. The best model for each was mapped in QGIS, and reclassified into 5 categories: 0 to 0.2 (probability very low); 0.2 to 0.4 (probability low); 0.4 to 0.6 (moderate); 0.6 to 0.8 (high) and 0.8 to 1 (very high) (Namgyal et al. 2021).

In order to mask out areas recently not used for agriculture, we use the land cover map of Ecuador (Ministerio de Agricultura y Ganadería [MAG] 2014) as in Table 3. This data from 2014 is the most recent version. We applied a 50% transparency mask so that areas that may become used for cattle raising in the future are included, even though prediction accuracy may be lower in unsampled areas.

**Table 3:** Columns of land use Ecuador shape use to overlaid to RF models

Land use	Included or excluded
Agricultural	Excluded
Anthropic	Included
Agricultural association	Excluded
Forest	Included
Water bodies	Included
Other areas	Included
Shrub vegetation	Included
Farming	Included
Farming conservation	Included
Forest agriculture and farming	Excluded
Mixed agriculture and farming	Excluded
Conservation and protection	Included
Forestry	Included
Livestock	Excluded
Livestock conservation	Excluded
Unproductive land	Included

Finally, we made a map of the probability of having both species by combining the suitability maps of *R. microplus* and *A. cajennense* s.l. Suitability values were reclassified as: 0 to 0.5 = 0, and 0.51 to 1 = 1. The reclassified maps were combined in a new raster with the following classes: 0 = no ticks, 1 = one tick species, and 2 = two tick species.

## Results

### Presence data

2895 farms were included, of which 1780 farms had *R. microplus* presence. 460 farms had *A. cajennense* s.l., and 378 both species (Table 4).

**Table 4:** Presence and absence of *Rhipicephalus microplus*, and *Amblyomma cajennense* sensu lato, in cattle farms per province

Provinces	Farms sampled	*R. microplus*				*A. cajennense* s.l			
		Absence		Presence		Absence		Presence	
	#	#	%	#	%	#	%	#	%
Azuay	142	142	100	0	0	142	100	0	0
Bolivar	241	196	81	45	19	234	97	7	3
Carchi	81	72	89	9	11	80	99	1	1
Chimborazo	276	270	98	6	2	276	100	0	0
Cotopaxi	220	194	88	26	12	214	97	6	3
Imbabura	144	105	73	39	27	142	99	2	1
Loja	332	168	51	164	49	269	81	63	19
Pichincha	127	62	49	65	51	127	100	0	0
Tungurahua	199	195	98	4	2	199	100	0	0
El Oro	86	15	17	71	83	56	65	30	35
Esmeraldas	98	40	41	58	59	24	24	74	76
Guayas	137	54	39	83	61	98	72	39	28
Los Rios	110	32	29	78	71	71	65	39	35
Manabi	312	112	36	200	64	150	48	162	52
Santa Elena	17	8	47	9	53	8	47	9	53
Sto. Domingo	111	14	13	97	87	85	77	26	23
Morona Santiago	7	0	0	7	100	7	100	0	0
Napo	79	21	27	58	73	79	100	0	0
Orellana	9	0	0	9	100	9	100	0	0
Pastaza	52	51	98	1	2	52	100	0	0
Sucumbios	30	2	7	28	93	30	100	0	0
Zamora Chinchipe	85	27	32	58	68	83	98	2	2
Total	2895	1780	61	1115	39		84	460	16

### Distribution maps

**Fig. 2:**
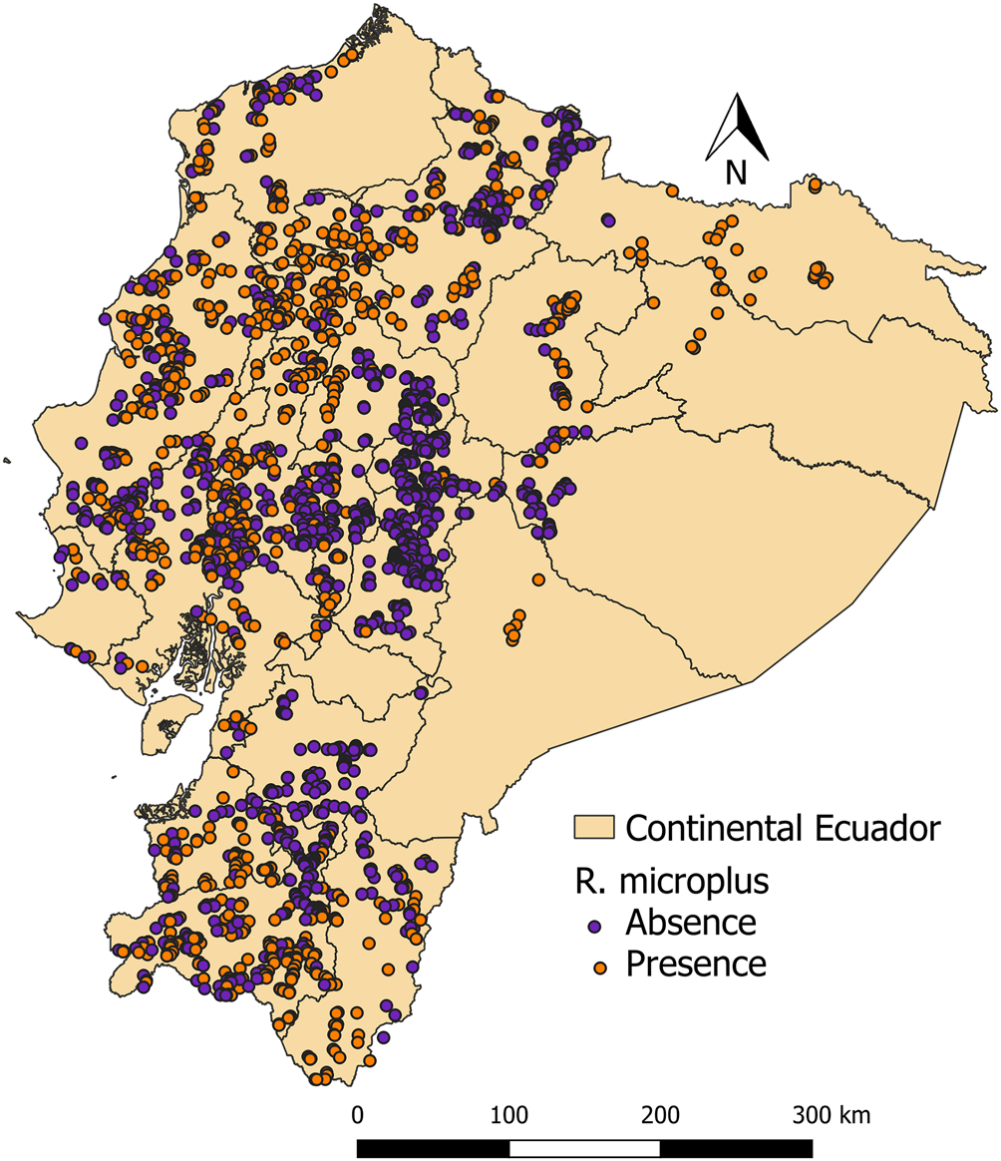
Distribution map of *Rhipicephalus microplus* at farm level in continental Ecuador

**Fig. 3:**
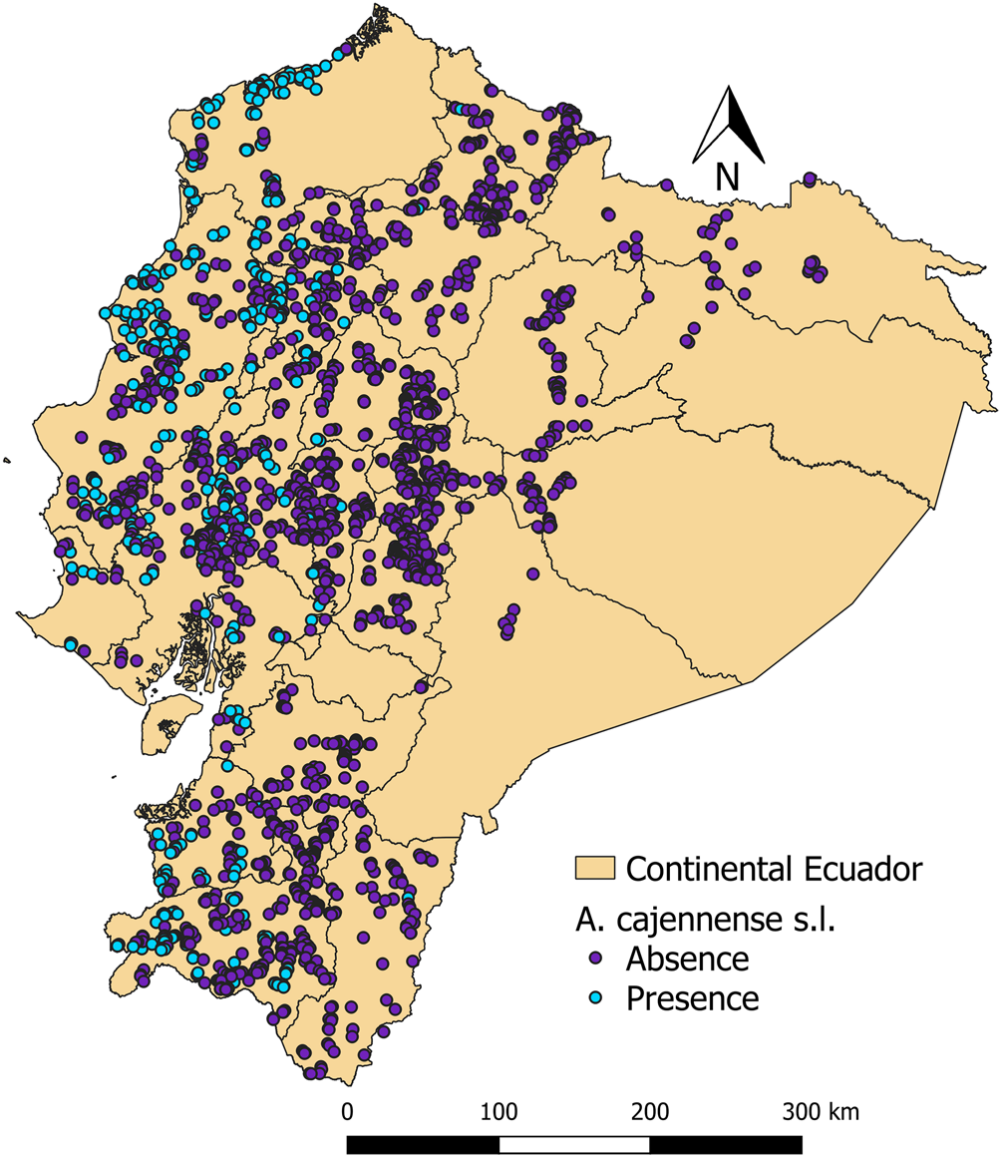
Distribution map of *Amblyomma cajennense* sensu lato at farm level in continental Ecuador

**Fig. 4:**
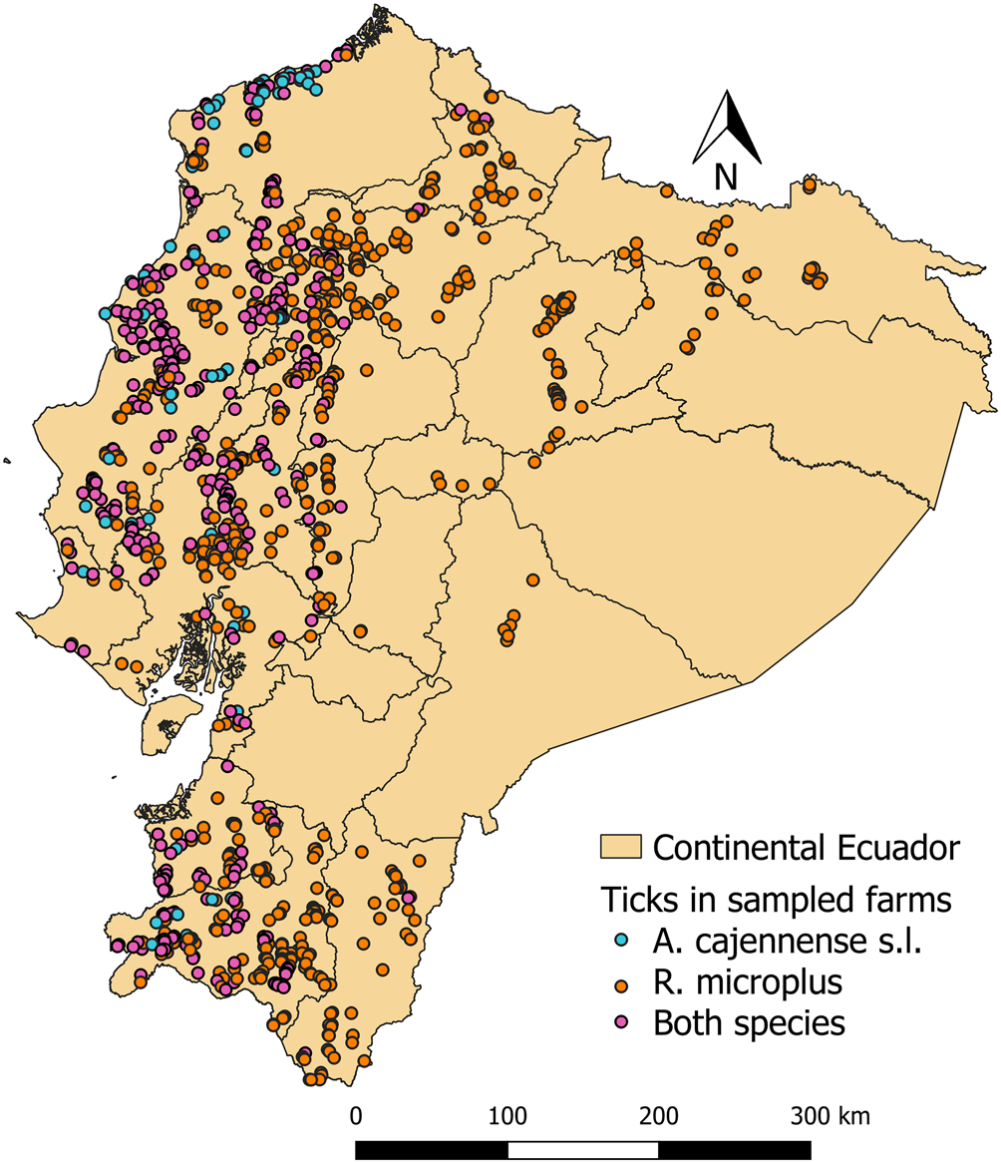
Distribution map of *Rhipicephalus microplus*, and *Amblyomma cajennense* sensu lato co-occurrence at farm level in continental Ecuador. The points were jittered to avoid overlap

The distribution of *Rhipicephalus microplus* and *Amblyomma cajennense* sensu lato. is presented in Figs. 2 and 3. Farms with presence of one and both are shown in Fig. 4. *R. microplus* is widely distributed in the Coastal, foothills of the Andean zone and Amazon. *A. cajennense* s.l. is distributed in the Coastal zone and in the western foothills of the Andean zone.

### Bi-variable generalized linear models

The individual association between bioclimatic factors and the presence of *R. microplus* and *A. cajennense* s.l. showed P values lower than 0.2 for all variables tested (Table 5).

**Table 5:** P value and Odds ratio for the bi variable GLM of *Rhipicephalus microplus,* and *Amblyomma cajennense* sensu lato in association with bioclimatic variables obtained from CHELSA

Variables		*R. microplus*		*A. cajennense* s.l	
		P	ODDS ratio	P	ODDS ratio
Bio	1	< 0.01	1.2694	< 0.01	1.4823
Bio	2	< 0.01	0.5649	< 0.01	0.5965
Bio	3	< 0.01	0.9423	< 0.01	0.9681
Bio	4	< 0.01	1.0076	< 0.01	1.0123
Bio	5	< 0.01	1.2966	< 0.01	1.5575
Bio	6	< 0.01	1.2429	< 0.01	1.4146
Bio	7	< 0.01	0.6338	< 0.01	0.6376
Bio	8	< 0.01	1.2568	< 0.01	1.4602
Bio	9	< 0.01	1.2799	< 0.01	1.4819
Bio	10	< 0.01	1.2546	< 0.01	1.4409
Bio	11	< 0.01	1.2811	< 0.01	1.5295
Bio	12	< 0.01	1.0003	< 0.01	0.9997
Bio	13	< 0.01	1.0037	< 0.01	1.0010
Bio	14	< 0.01	0.9983	< 0.01	0.9636
Bio	15	< 0.01	1.0237	< 0.01	1.0456
Bio	16	< 0.01	1.0012	< 0.01	1.0003
Bio	17	0.06	0.9997	< 0.01	1.0003
Bio	18	< 0.01	1.0011	< 0.01	1.0003
Bio	19	0.09	0.9997	< 0.01	0.9914
VPD	Max	< 0.01	1.0039	< 0.01	1.0051
VPD	Min	< 0.01	1.0045	< 0.01	1.0060
VPD	Mean	< 0.01	1.0043	< 0.01	1.0057

*P*  P value

### Multivariate generalized linear model

The final explanatory GLM after removing colinear variables, and with the lowest AIC for *R. microplus* and *A. cajennense* s.l., included 10 bioclimatic variables: Bio1, Bio2, Bio3, Bio4, Bio12, Bio13, Bio14, Bio18, VPD_max, and VPD_min (Table 6).

**Table 6:** Multivariate generalized linear models of the presence of cattle ticks, *Rhipicephalus microplus*, and *Amblyomma cajennense* sensu lato and their association with bioclimatic factors

Models		*R. microplus*		*A. cajennense* s.l	
Variables		P	ODDS ratio	P	ODDS ratio
Bio	1	< 0.01	39.38 (24.09–65.34)	< 0.01	359.30 (138.17–1014.14)
Bio	2	< 0.01	1.65 (1.35–2.02)	< 0.01	1.95 (1.49–2.55)
Bio	3	< 0.01	0.65 (0.55–0.77)	< 0.01	0.53 (0.41–0.68)
Bio	4	< 0.01	0.63(0.53–0.74)	< 0.01	0.55 (0.44–0.69)
Bio	12	< 0.01	8.10 (3.80–17.41)	< 0.01	17.14 (4.14–72.24)
Bio	13	< 0.01	0.43 (0.244–0.75)	< 0.01	0.15 (0.05–0.38)
Bio	14	< 0.01	0.35 (0.22–0.53)	< 0.01	0.06 (0.02–0.16)
Bio	18	< 0.01	0.39 (0.28–0.56)	< 0.01	0.47 (0.29–0.78)
VP	Max	< 0.01	0.12 (0.07–0.21)	< 0.01	0.04 (0.02–0.08)
VP	Min	0.01	1.84 (0.16–2.95)	< 0.01	3.07 (1.65–5.79)

*P*  P value

The AUC-ROC, sensitivity, and specificity are above 0.68 for both models (Table 7).

**Table 7:** Values of the area under the curve (AUC), specificity, and sensitivity for the multivariate generalized linear models

Model	AUC-ROC	Specificity	Sensitivity
*R. microplus*	0.8360	0.6865	0.8547
*A. cajennense* s.l	0.9009	0.7967	0.8957

### Predictive model

**Fig. 5:**
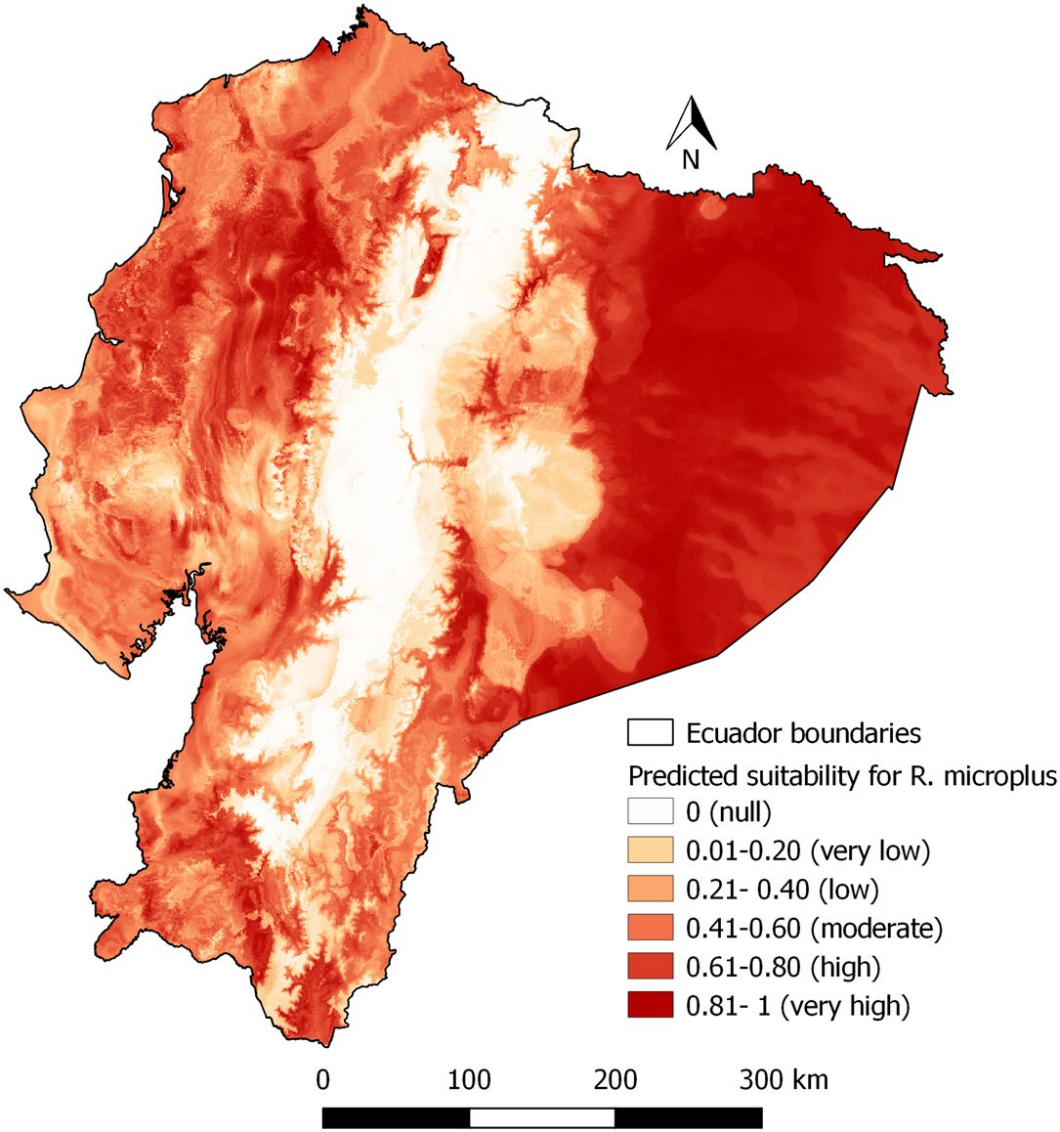
Predicted suitability for *Rhipicephalus microplus*

**Fig. 6:**
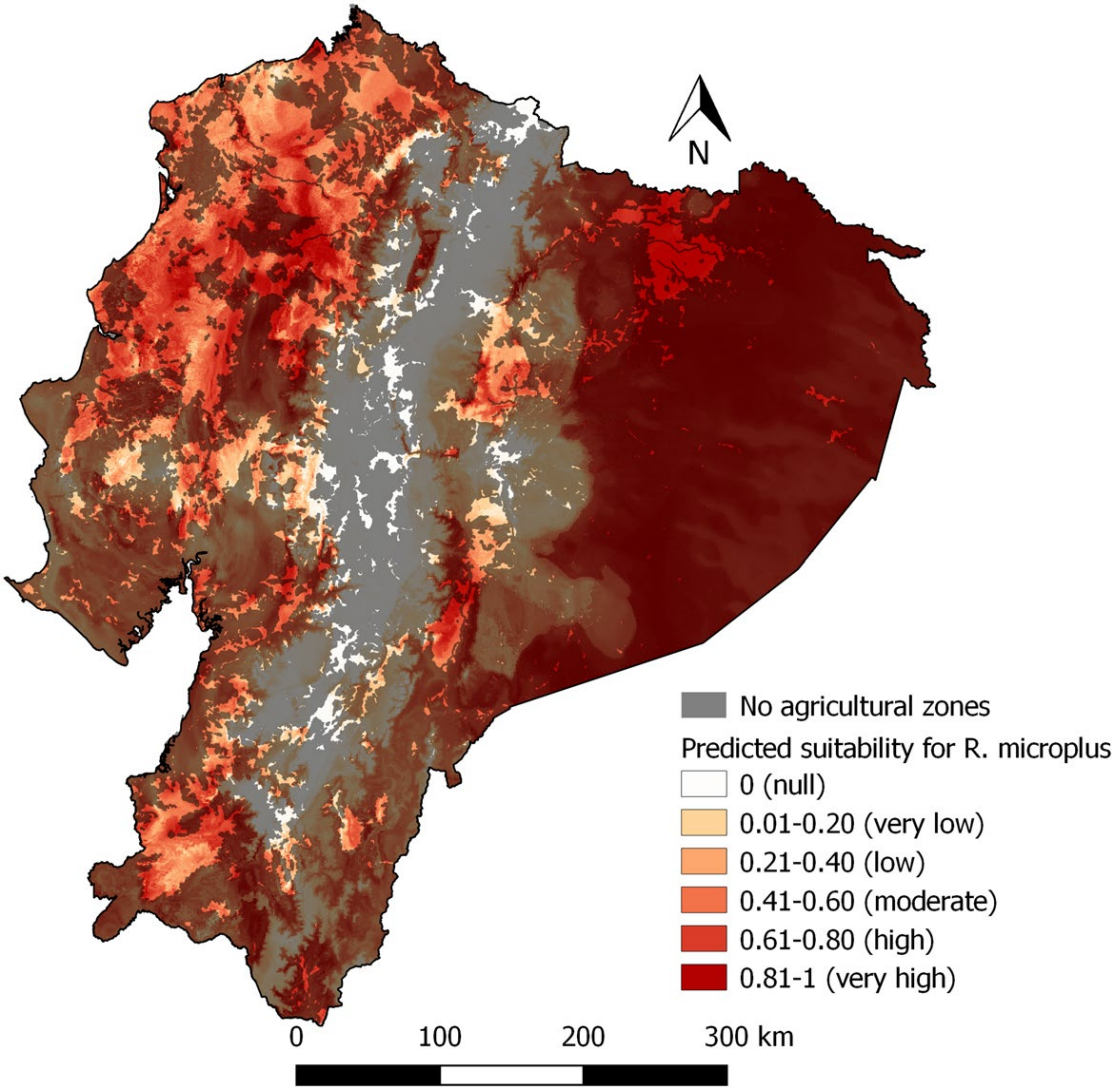
Predicted suitability for *Rhipicephalus microplus* with areas without agricultural activities masked out with a 50% transparency mask

**Fig. 7:**
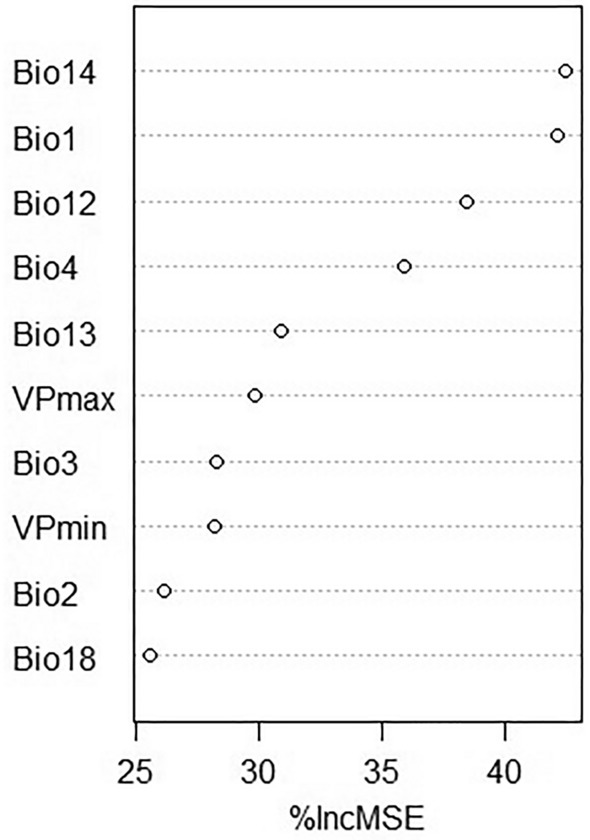
Values of Percentage Increase in MSE (Regression) (%IncMSE) for the habitat suitability model RF for *Rhipicephalus microplus*

Figure 5 shows the potential distribution of *R. microplus* obtained by RF. The Amazon and Coastal zones are highly suitable, while only some Andean areas were suitable, particularly in Andean valleys and foothills. Figure 6 is showing the suitability map for *R. microplus* with areas where cattle is currently absent masked out. For *R. microplus* the highest value of Percentage Increase in MSE (Regression) (%IncMSE) corresponds to Bio 14 followed by Bio 1 with 42.44 and 42.18 respectively (Fig. 7). Bio 4 and Bio 12 were also important variables. Thus, a combination of factors like temperature, humidity, and reduced change in seasonality were determinant factors for *R. microplus* presence.

**Fig. 8:**
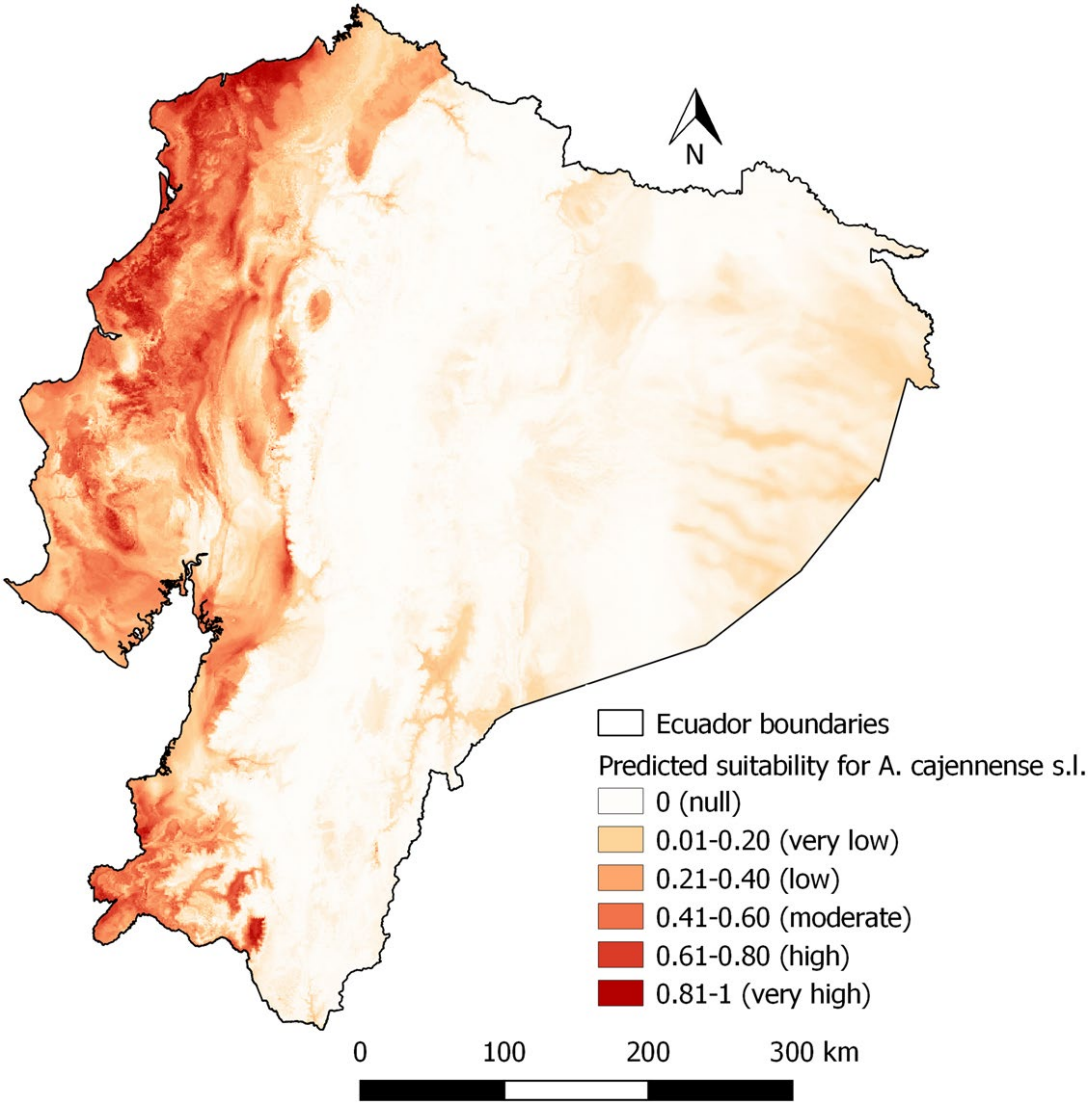
Predicted suitability for *Amblyomma cajennense* sensu lato

**Fig. 9:**
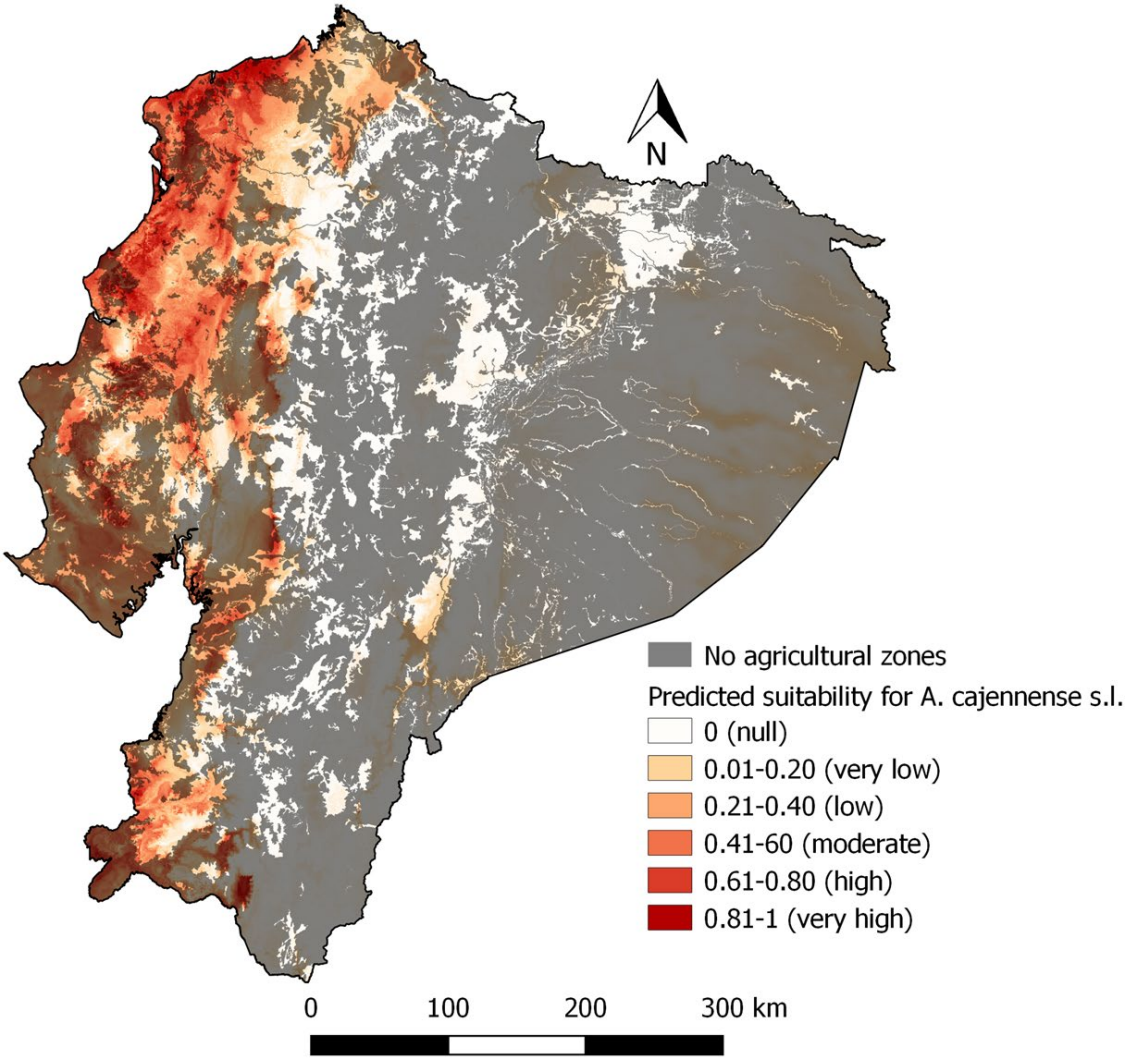
Predicted suitability for *Amblyomma cajennense* sensu lato with areas without agricultural activities masked out with a 50% transparency mask as a scenario

**Fig. 10:**
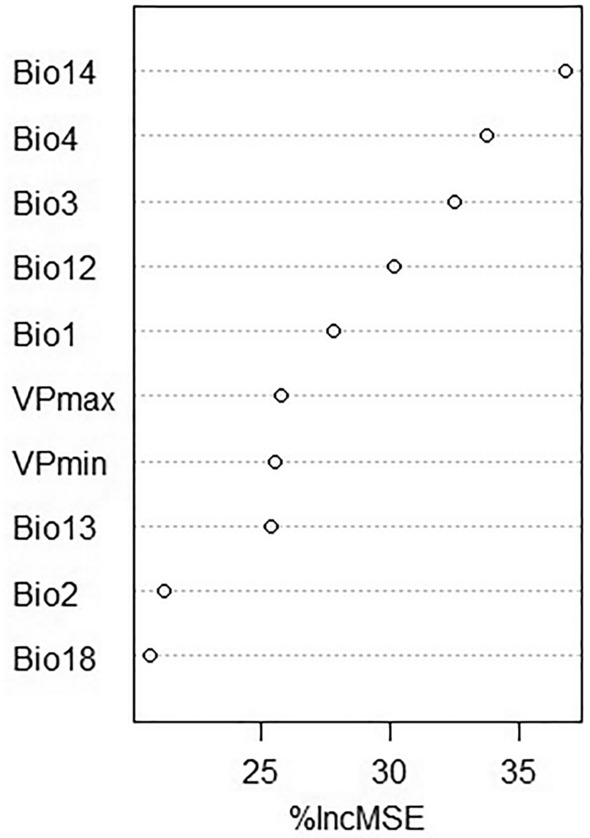
Values of Percentage Increase in MSE (Regression) (%IncMSE) for the habitat suitability model RF for *Amblyomma cajennense* sensu lato

Areas with high predicted suitability for *A. cajennense* s.l. are all found in the Coastal zone (Fig. 8). Predicted suitability is low in the Andean and Amazon zones. However,the foothills of the western cordillera also have a degree of predicted suitability. Figure 9 presents the suitability with non-agricultural areas masked out. With respect to the importance of the variables, the highest %incMSE values correspond to Bio14 and Bio4 with 36.74 and 33.71 respectively (Fig. 10).Areas with marked seasonality, reduced isothermality are related to *A. cajennese* s.l. presence.

**Fig. 11:**
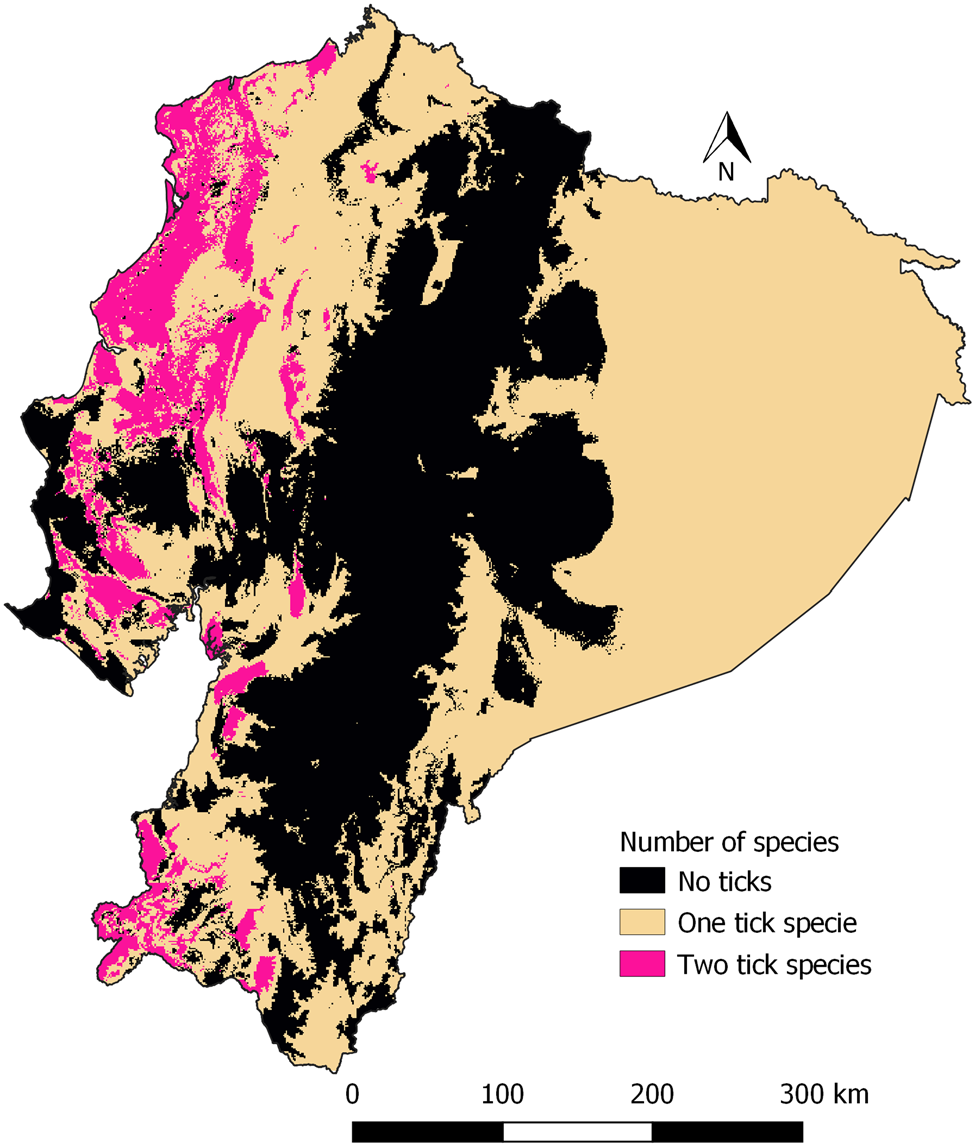
Combination of predicted suitability over 0.5 for both Rhipicephalus microplus, and *Amblyomma cajennense* sensu lato

All provinces of the Coastal zone have a high suitability for both tick species, as well as Loja province in the Andean zone (Fig. 11).

All accuracy metrics for the Random Forest models were above 0.93 (Table 8).

**Table 8:** Accuracy, sensitivity, specificity, and kappa for the Random forest models for *Rhipicephalus microplus,* and *Amblyomma cajennense* sensu lato

Model	Accuracy	Sensitivity	Specificity	Kappa
*R. microplus*	0.968	0.970	0.966	0.933
*A. cajennense* s.l	0.983	0.992	0.935	0.937

The models were also trained and tested with 80% and 20% of the entire data, respectively. The average of 10 random models is presented in Table 9.

**Table 9:** Average of accuracy metricts for 10 random cross validated models for Rhipicephalus microplus, and *Amblyomma cajennense* sensu lato

Model	Accuracy	Sensitivity	Specificity	Kappa
*R. microplus*	0.778	0.797	0.756	0.539
*A. cajennense* s.l	0.878	0.925	0.634	0.569

## Discussion

The results of this study highlight the widespread distribution of *R. microplus* and *A. cajennense* s.l. ticks in tropical and subtropical areas of Ecuador, as supported by multiple studies (Escobar et al. 2015; Bustillos and Rodríguez 2016; Rodríguez-Hidalgo et al. 2017; Maya-Delgado et al. 2020; Chávez-Larrea et al. 2021; Guglielmone et al. 2021; Paucar et al. 2022; Pérez-Otáñez et al. 2023). Earlier studies had identified isolated presence of *R. microplus* in the provinces of Los Ríos (Escobar et al. 2015), Pichincha, Tungurahua, Manabí (Diazalulema 2015; Rodríguez-Hidalgo et al. 2017; Chávez-Larrea et al. 2021), Napo, Sucumbíos, Orellana (Quezada and Quezada 2015; Insuaste Taipe 2021). *A. cajennense* s.l. was documented in El Oro (Nava et al. 2014), Pichincha and Manabí (Beati et al. 2013; Paucar et al. 2022). However, this information is scattered and needs to be assembled to get a consolidated understanding of the distribution of these species in continental Ecuador.

In this study, we assembled various datasets to build a complete picture of the current distribution of *R. microplus* and *A. cajennense* s.l. in continental Ecuador, and we evaluated associations with bioclimatic variables to build a spatially continuous habitat suitability map.

Climate conditions, particularly temperature, humidity, and precipitation, were found to be crucial factors influencing the distribution and development of ticks, which is in line with previous research (Pfäffle et al. 2013). For this study, bioclimatic variables like Bio1, Bio2, Bio3, Bio4, Bio12, Bio13, Bio14, Bio18, VP_min, and VP_max were highly significant in the GLM, with Bio1, Bio12, Bio14 and VPD variables having the highest (above 1) and lowest (below 1) odds ratio. This is coherent with Marques et al. (2020) who in their study determined that the bioclimatic variables from WorldClim, such as Bio1, Bio4, Bio12, Bio14, and relative humidity, are useful predictors for *R. microplus*. Likewise, Bio 18 contributed to the models developed by Namgyal et al. (2021) in addition to elevation and land cover, which were not evaluated in this study as we focused on climatic factors. These results are coherent with tick biology for both species studied (Estrada-Peña et al. 2006c, 2014; Pascoe et al. 2019). In the case of *A. cajennense* s.l., the study of Aguilar-Domínguez et al. (2021) with *A. mixtum*, a species belonging to the *cajennense* complex, showed four important bioclimatic factors: Bio4, Bio6, Bio7 and Bio12, of which Bio4, and Bio12 are coherent with this study. *A. cajennense* s.l. has a suitable habitat in the coastal zone of Ecuador where seasonality of precipitations is marked but limited for temperature. Thus, is thermality played an important role in the distribution of *A. cajennense* s.l.

The present study utilized a Random Forest model to predict suitable areas for *R. microplus* and *A. cajennense* s.l. based on bioclimatic factors. Random Forest was used because we have both presence and absence data. In West Africa, Zannou et al. (2022) tested several models and found that Random Forest was an accurate model for habitat suitability of *R. microplus*.

Our models suggest that *R. microplus* could potentially occur in most areas of Ecuador, except the Andes Mountains, with the Amazon and Coastal zones had high suitability. Ecuador’s diverse ecological formations associated with varied microclimates create an intricate landscape for tick distribution (Galeas et al. 2013). However, caution is needed when considering areas that are currently unused for agricultural or livestock purposes, and as a result cannot be sampled for ticks dependent on cattle (Fig. 6 and 9), like the Amazon. Our model predicted suitability in those areas, suggesting tick may rapidly become a problem if those forest areas are cleared for pasture and cattle introduction. However, we focused on climatic determinants of tick abundance, and could not account for cattle, a factor necessary for tick presence. While the Amazon zone is known for its extensive forested regions and 16 Natural Protected Areas spanning 30,514 km2 (López. et al. 2012; Galeas et al. 2013), it is facing increasing pressure from anthropic activities, such as oil extraction, mining, deforestation, road construction, colonization, and disorganized rural settlements. Large forested habitats are becoming fragmented landscapes, potentially leading to changes in tick distribution (López et al. 2012; Galeas et al. 2013; Alemán Gaínza et al. 2014; Cicuttin 2019; Vale et al. 2019; Galeas et al. 2013). Access to accurate cattle distribution data, currently unavailable at a fine enough resolution in Ecuador, is a must for *R. microplus*. The question is different *Amblyomma* ticks, which parasite diverse wild hosts as well as cattle. Some such hosts are abundant in the Amazon zone abundant, such as mammals, birds, reptiles, and amphibians live. They serve as hosts for other species of ticks such as *Amblyomma latepunctatum* Tonelli Rondelli, *Amblyomma humerale* Koch, *Amblyomma dissimile* Koch, etc. (Guglielmone et al. 2021). Should the Amazon undergo extensive deforestation for cattle husbandry, the model may need to be updated locally for the association changes in climate conditions, including increased temperatures and reduced humidity (Pfäffle et al. 2013). As cattle mobility has been identified as a primary factor in the spread of cattle ticks (Chávez-Larrea et al. 2021), if cattle husbandry expands in the Amazon, great vigilance for tick issue should be applied, and surveillance started early.

The study’s findings diverge from some previous research. In our study, the Amazon zone and Coastal zone are highly suitable, as well as Andean valleys, and the eastern and western foothills of the Andes for *R. microplus*. This differs from results obtained at the regional level, for example, by Marques et al. (2020) who report high suitability in the Andean zone and medium suitability in the Coastal zone and the Amazon zone. Estrada-Peña (1999) showed Ecuador as non-suitable for *R. microplus.* In addition (Estrada-Peña et al. 2005) show only the northwestern part of the country (Esmeraldas and Carchi) as zones with high suitability in 1999, the northern provinces of the three regions, and parts of the southern zone as suitable zones in 2025 and 2050. This study provides a good estimation of the habitat suitability, with sensitivity of 0.97 and specificity of 0.96, similar values to Estrada-Peña (1999) with sensitivity of 0.91 and specificity of 0.88. Overall, our sample covers the diverse Ecuadorian environment and its specificities much more exhaustively.

For *A. cajennense* s.l., the model in this study shows that the highly suitable areas are limited to the Coastal zone and areas near to the western foothills of the Andes. To compare the results of this study, we will focus on studies conducted on *Amblyomma mixtum*, which is part of the *Amblyomma cajennense* s. l. complex. This choice is based on the description by other authors, suggesting that *A. mixtum* is the species present in Ecuador (Alcala-Canto et al. 2018; Pascoe et al. 2019; Aguilar-Domínguez et al. 2021). The model for *Amblyomma mixtum* Koch proposed by Nava et al. (2014) shows also highly suitability for the Coastal zone, similar to this study. Aguilar-Domínguez et al. (2021) describe the potential distribution of *A. mixtum* and show the Coastal zone of Ecuador as suitable as well, and for the coming 50 years. Our study also shows a very low suitability in some areas of the Amazon where the presence of this species has not been reported and most of them correspond to non-livestock (agricultural) areas. *A. mixtum* is known from western Ecuador (provinces of El Oro, Guayas, Los Ríos, Manabí and Pichincha) (Orozco Álvarez 2018; Paucar et al. 2022), *A. cajennense* s.l. was found in various environments, including dry and semiarid areas as well as riparian forests and savanna lowlands. (Estrada-Peña et al. 2014). However, its survival within these habitats is contingent on specific microclimatic conditions, particularly a relative humidity not dropping below 80% for extended periods (Pfäffle et al. 2013). *A*. *cajennense* s.l. spend more time and energy in finishing its life cycle because its way to feed (Polanco Echeverry and Ríos Osorio 2016). Due to species-specific differences, the abundance of *Rhipicephalus microplus* surpasses that of *Amblyomma cajennense* s.l. in the studied farms. This can be explained by the one-host cycle of *R. microplus* and its hostspecificity. *R. microplus* is also well adapted to the tropical and subtropical climate of Ecuador. Over 80% of farms surveyed had *R. microplus*, whereas *A. cajennense* s. l. accounts for only approximately 15% of farms infested the total tick population in these farms.

Figure 11 highlights areas where both tick species are likely to coexist on the same farm, all of the 43 farms surveyed by Alonso-Díaz et al. (2013) in Mexico had both species present. We found that 378 out of 2895 farms had both species, primarily in the Coastal zone and western foothills of the Andean Mountains. *R. microplus* and *A. cajennense* s.l. are closely associated with their respective hosts, with *R. microplus* primarily infesting cattle and *A. cajennense* s.l. primarily infesting equines, but also cattle (Guglielmone et al. 2021).

The model operates at the national scale and gives the broad spatial trends in suitability, but at the fine scale, other factors will determine tick presence and abundance. At the farm level, factors such as the presence and abundance of hosts (cattle), as well as control practices, such as use of acaricide, cattle resistance to ticks, and organization of grazing systems, play a significant role in the distribution and abundance of cattle ticks (Estrada-Peña et al. 2005; Alemán Gaínza et al. 2014; Paucar et al. 2022). Local models may be able to capture such factor, that are poorly documented at the national scale.

This research is the first to provide a national-level assessment of tick distribution in continental Ecuadorian, offering valuable insights into tick presence and absence across the country. This comprehensive understanding of tick distribution can aid in the development of effective tick control and management plans, considering the differences in tick species, their distribution, and their biological characteristics.

The study emphasizes the importance of continuous research to monitor tick populations as tick distribution may change over time due to factors including climate change and human activities. Moreover, knowing the main tick species and their spatial distribution is crucial for developing targeted strategies to mitigate tick-related problems and protect livestock health and productivity. The study’s findings call for the implementation of tick prevention/control plans considering the specific ecological contexts and host interactions, which could help reduce the negative impacts of ticks on livestock farming. Moreover, it is crucial to consider diverse approaches for their prevention/control due to the broad host range of *A. cajennese* s.l., and the specificity of *R. microplus* for cattle. Additionally, it highlights the importance of addressing the challenges posed by *R. microplus* resistance to acaricides, which has become a significant concern for livestock farmers in Ecuador (Rodríguez-Hidalgo et al. 2017; Maya-Delgado et al. 2020; Paucar-Quishpe et al. 2023).

In conclusion, this study significantly contributes to the understanding of tick distribution in Ecuador, shedding light on the climatic factors influencing their presence and abundance. The results offer valuable insights for policymakers, farmers, and researchers to develop effective tick preventive/control plans and protect livestock health in the region. However, continuous research and monitoring are necessary to keep abreast of the evolving tick distribution patterns and make informed decisions for tick management and livestock production in the future.

## Data Availability

The data that support the results of this study are available from the corresponding author upon request.
